# Development of an Intervention for Managing Adolescent Anxiety Using Community-Based Participatory Methods: Protocol

**DOI:** 10.2196/75501

**Published:** 2025-10-31

**Authors:** Ijeoma Opara, Cristina Pagan, Raquel E Rose, Beatriz Duran-Becerra, Catherine Mwai, Shreya Jadhav, Hailey Valles, Kammarauche Aneni

**Affiliations:** 1 Yale School of Public Health, Yale University, New Haven, CT, United States; 2 Safe Counsel, LLC, Paterson, NJ, United States; 3 Yale School of Medicine, Yale University, New Haven, CT, United States; 4 The Child Study Center, Yale University, New Haven, CT, United States

**Keywords:** adolescent mental health, mental health, urban, prevention, community, community-based participatory research, co-development, co-design

## Abstract

**Background:**

Youth of color who live in urban communities face disproportionate anxiety levels due to systemic inequities, including exposure to violence, economic instability, and neighborhood disadvantage. Despite increased need, these communities often lack accessible mental health interventions.

**Objective:**

This study presents a protocol for an anxiety prevention intervention developed through a community-based participatory approach that is tailored to urban youth of color using community-based participatory research methods.

**Methods:**

The intervention, co-developed with a community partner and guided by a youth advisory board, includes 5 structured weekly sessions on psychoeducation, coping skills, and role-playing exercises. Facilitators trained in social work or psychology will deliver the intervention, with at least 1 facilitator from the target community ensuring cultural relevance. Recruitment will occur through collaboration with a local high school, with counselors identifying high-risk youth. We will enroll a sample of 30 high school–aged youth at minimum (maximum 50 youth) into the study. Two cohorts of youth will participate in the study. The groups will be separated by sex (male and female). The primary outcome is reduction in anxiety, measured by the Generalized Anxiety Disorder-7 scale. Data will be collected at baseline, after the intervention, and during follow-up assessments (3 months after). Statistical analyses will include parametric tests (eg, repeated measures ANOVA and 1-tailed *t* tests) to compare anxiety reduction across groups.

**Results:**

This pilot intervention is a part of a larger study that began in September 2020 and ended in August 2025. Enrollment for the pilot intervention began in May 2025. The anxiety intervention is expected to reduce anxiety among a high-risk group of youth. Methods to improve facilitator fidelity to the intervention model are expected to support high fidelity to the curriculum.

**Conclusions:**

This study highlights the development of a new anxiety intervention using a community-based participatory approach. Findings will be reported and used to scale up the pilot intervention into a larger clinical trial to serve a larger population of youth in the targeted community. In addition, the results will contribute to knowledge on improving mental health accessibility for marginalized youth. If effective, this model could be expanded to support youth in other underresourced communities.

## Introduction

### Background

In the United States, the prevalence of anxiety among youth has continued to steadily rise over the past few years [1]. Findings from the National Survey of Children’s Health showed a 27% increase in anxiety from 2016 to 2019 and, by 2020, 5.6 million children had been diagnosed with anxiety problems [1]. Increased anxiety among youth of color has also been observed over the past few years [2,3]. Addition, the rate of increase in anxiety disorders among Black people overall exceeded the rate seen for White people over the same period (17.1% for Black people vs 4.5% for White people) [2]. Moreover, data from the National Comorbidity Survey, which assessed for lifetime prevalence of mood and anxiety disorders among English-speaking households, revealed that Hispanic or Latinx youth were at the highest risk for mood and anxiety disorders compared to non-Hispanic White adolescents [3]. Besides disparities across racial and ethnic groups, adolescents from low-income households, especially those in urban communities, are at higher risk for anxiety compared to their peers from high-income households [4,5].

While there may be many factors that play a role in the development of anxiety symptoms among youth, research has shown that increased rates of mental health issues, specifically among urban youth, are significantly related to the environment in which they live and if they are at a neighborhood disadvantage [6]. Characteristics of the urban environment, such as community violence, gang activity, substance use, and poverty, disproportionately affect urban youth and increase their exposure to potentially traumatic events such as incidents related to community-level violence [7]. This type of trauma exposure has been linked to the development of depression, anxiety, and early-age substance use. Similarly, neighborhood disadvantage consisting of limited resources, lack of safety, subpar housing, and unemployment has also been associated with anxiety among urban youth [5,7]. As evidenced, due to the amount of stressors youth are exposed to in urban communities, anxiety among this population is a significant public health problem, and the lack of resources in these communities exacerbates these issues. Underresourced urban communities are in dire need of services and programs that can improve the climates of spaces youth spend the majority of their time in, which can improve the overall mental health of the youth living in these communities.

As youth spend approximately one-third of their early developmental years within an academic setting, schools have been identified as effective sites for the implementation of mental health programming [8,9]. Due to schools being such key places for youth, school-based interventions have the opportunity of reaching youth who may have been previously unidentified or untreated [10]. Compared to community-based mental health clinics and primary care settings, delivering interventions in urban school settings, as an example, can address some of the many barriers faced by urban youth in underresourced communities such as removing the need for travel, unfamiliarity, cost, and time [11].

### Importance of Community-Based Participatory Research

Community-based participatory research (CBPR) can be used as a critical approach in the development and implementation of mental health interventions, particularly in urban communities, as it is essential that these programs and interventions incorporate the voices of those living in these communities [12]. Researchers agree that the insights gained from receiving direct feedback from community members enhance the researchers’ understanding of the problems affecting the community while also building on the strengths of the community. CBPR engages community members as equal partners and cogenerators of knowledge throughout the research process, ensuring that interventions are culturally relevant, contextually appropriate, and responsive to the specific needs of the population [12]. This collaborative methodology has proven effective in addressing systemic issues that contribute to mental health disparities, such as poverty, exposure to violence, and inadequate access to health care services [13]. Furthermore, CBPR fosters trust between academic researchers and historically marginalized communities, promoting the sustainability of mental health programs and enhancing their real-world impact [14].

Youth advisory boards (YABs) within CBPR projects, composed of young individuals from the very communities being studied, with diverse perspectives, play a pivotal role in ensuring that interventions are youth centered, engaging, and accessible. In recent years, research has shown the importance of including youth feedback in the development of mental health interventions for them [15]. It has been shown that the use of YABs helps to ensure that youth voices are heard within systems and helps to identify youth priorities related to mental health needs and issues [16]. Research also demonstrates that the use of youth participatory methods is helpful in obtaining meaningful insight from youth, engages them in the research process, and therefore empowers youth to be coparticipators in problem solving [17]. On a larger scale, including youth participation in research can also lead to more effective community-level policies that directly improve health outcomes and quality of life of this population [18]. By having youth actively participating in the co-design process, YAB members provide valuable insights into the language, delivery methods, and intervention components that resonate with their peers. Their involvement fosters a sense of ownership and empowerment, increasing the likelihood of program uptake and success.

Pertaining to youth mental health outcomes, engaging community members and YABs is crucial in developing effective interventions for young people, particularly in addressing mental health challenges such as anxiety. Community members offer invaluable insights into local needs, cultural dynamics, and systemic barriers that can influence the implementation and sustainability of interventions [18]. Their lived experiences help shape programs that are contextually relevant and responsive to the specific challenges faced by youth in their communities.

### Purpose of This Study

The purpose of this protocol is to outline steps that were taken for the development of a pilot feasibility trial of a community-based psychoeducational intervention for anxiety designed to alleviate anxiety symptoms among youth living in an urban environment. This curriculum was developed through a collaborative partnership with a licensed clinical social worker who was born and raised in the study setting, ensuring that the intervention is both culturally relevant and grounded in the lived experiences of the community. Recognizing the unique stressors faced by youth in urban settings, including exposure to community violence, economic instability, and limited access to mental health resources, this program integrates evidence-based anxiety prevention strategies with community knowledge. The curriculum is designed to be interactive, incorporating cognitive behavioral techniques, mindfulness practices, and resilience-building exercises tailored to the realities of youth living in this community. By centering local expertise and youth voices, this intervention seeks to empower participants with the tools needed to manage stress, build emotional resilience, and improve overall mental well-being, while fostering a sense of community support and engagement. The primary aims of this intervention are to (1) increase participants’ knowledge and understanding of anxiety, including its causes, symptoms, and physiological effects; (2) reduce stigma by normalizing conversations around anxiety and seeking different levels of help (eg, peer support, spiritual support, talking to a trusted adult, and therapy); and (3) increase participants’ confidence in managing anxiety by equipping them with practical coping skills to navigate stressors in their daily lives.

## Methods

### Study Setting

The study will be conducted in Paterson, New Jersey, a northeastern urban community with a population of approximately 157,864 residents, with children and youth aged <18 years comprising about 27% of the population [19]. The city faces significant socioeconomic challenges, including a poverty rate of 23.5%, more than double the state average of 9.7%. These economic hardships contribute to increased mental health concerns among residents, particularly youth, highlighting the urgent need for targeted mental health interventions. The study’s principal investigator (PI) has worked with the city as a community-based participatory researcher for almost a decade and leads multiple National Institutes of Health (NIH)–funded studies centered in this community, including a 5-year NIH-funded grant, the Paterson Prevention Project, which examines the impact of neighborhoods on youth substance use and mental health in the city [20]. Using previously published research from work conducted in the target community, and using a community-based participatory approach, the study has identified anxiety as a critical concern among Paterson youth. Addressing anxiety through targeted interventions has emerged as a crucial step in improving mental health access and outcomes in this underserved community.

### Participatory Co-Design Process: Initial Development

The development of the intervention was a collaborative and community-engaged process that underscores the importance of co-designing with trusted community partners to enhance feasibility, cultural relevance, and sustainability. The initial intervention idea came from CP, a licensed clinical social worker and a longstanding, trusted community partner of the Paterson Prevention Project. Through her active participation in research activities in the project, including data collection and engagement with youth, CP identified a critical need for anxiety prevention resources and independently initiated an informal intervention at a local high school. Confirmed with evidence from qualitative and quantitative findings from the study, the PI decided to co-develop a pilot intervention with the community partner. This collaboration ensured that the intervention was not only informed by community insights but also grounded in evidence-based strategies. First, CP provided an outline of the sessions, such as topics that were important to address. CP met with the study’s PI and the study team several times to decide on a specific framework to use and activities that would be included in each session. By integrating evidence-based practices with the lived experiences and firsthand observations of a community-based mental health practitioner, the intervention was strengthened in its feasibility, acceptability, and potential for scalability.

Co-designing interventions with community partners such as CP facilitates the integration of scientific rigor with practical, real-world application, increasing the likelihood of successful implementation and long-term sustainability. This approach aligns with participatory research principles, which emphasize the value of local expertise in shaping interventions that are responsive to community needs. Through this iterative process, the intervention was refined to balance evidence-based strategies with culturally and contextually relevant components, ensuring it could be effectively scaled and sustained within the community.

### Theoretical Framework

The intervention is guided using cognitive behavioral therapy (CBT) principles, a well-established evidence-based approach that targets maladaptive thoughts and behaviors to improve mental health outcomes [21]. In addition to psychoeducation around symptoms of anxiety, CBT also emphasizes skill development and implementation such as breathing techniques, visualization, and graded exposure and role-playing. Given the unique needs of urban youth, adaptations of how CBT will be delivered have been developed to enhance its cultural relevance and effectiveness. Research has demonstrated that culturally tailored CBT interventions for urban youth populations incorporate contextual stressors such as systemic inequities, racial discrimination, and community violence, making the approach more applicable and impactful for these communities [22]. AdditionAdditionally, our intervention integrates principles from trauma-focused CBT, which has been shown to be effective in addressing trauma-related symptoms among youth who have experienced adversity [23]. By incorporating both frameworks alongside trauma-informed strategies, our approach aims to provide a comprehensive and culturally responsive intervention for urban youth facing multiple forms of psychosocial stressors.

### Development of the Intervention Facilitator Guide

#### Overview

The initial intervention materials for the curriculum were developed by CP, BD-B (project manager at the time of study), and the PI (IO) in collaboration with YAB members of the Paterson Prevention Project. The research staff was instrumental in the development of graphics and curriculum structure (SJ, CM, and HV); the accuracy of session topics and strategies was reviewed for accuracy by a licensed child psychiatrist on the team (KA) and postdoctora research fellow and psychologist (RER).

In addition, the Paterson Prevention Project YAB, which consists of 8 teenagers and young adults who live in Paterson, was instrumental in reviewing materials with research staff during biweekly meetings over the course of 2 years. All research activities including the development of sessions and evaluation materials were supervised and approved by the PI (IO). One of the advisory board members is a recent Master of Social Work graduate and has been trained as a cofacilitator of the intervention.

#### Theater Testing

Theater testing is a critical step in the development and refinement of an intervention, allowing facilitators to practice the delivery of the curriculum with a small group before full-scale implementation. This process helps identify strengths, areas for improvement, and any logistical or content-related challenges that may arise. By running the intervention in a controlled environment with a representative sample, facilitators can assess whether the activities, discussions, and pacing are engaging and effective for participants. Research supports the use of pilot or feasibility testing in intervention development, as it provides essential feedback to refine content, delivery style, and overall structure, ensuring that the program is both evidence based and practical for real-world application [24]. Additionally, theater testing allows facilitators to become more comfortable with the material, enhancing their ability to deliver sessions with confidence and consistency [25].

In February 2025, the study team conducted a theater test of the intervention with 5 members of our YAB during an 8-hour session in person in Paterson, New Jersey. The facilitators led participants through the full intervention while a designated study team member took notes on participant engagement, clarity of instructions, and areas needing improvement. After completing the session, the study team convened to discuss feedback and make necessary revisions to the curriculum. Additionally, a follow-up meeting was held with the PI to review the final adjustments before moving forward with pilot testing. This iterative process ensures that the intervention is responsive to the needs of the target population, aligns with best practices in youth mental health programming, and is optimized for successful implementation.

### Finalization of Intervention

While there are no control conditions, as the intervention will run over multiple iterations, a staggered start design will be used to account for potential differences in groups. A staggered start design is a phased implementation approach that allows for iterative assessment and refinement of an intervention before full-scale delivery. In this study, this design involves initiating the intervention with 2 initial cohorts, each consisting of 8 to 10 youth stratified by sex (male and female). This first cohort will complete sessions 1 to 5 while facilitators document process implementation using fidelity assessment tools. This approach enables real-time evaluation of intervention delivery, facilitator adherence, and participant engagement, allowing for any necessary refinements before subsequent cohorts are enrolled. Following the completion of the initial cohorts, an additional 2 cohorts will be recruited, with this process continuing until we reach our target enrollment of 30 to 50 youth. We anticipate recruiting 2 cohorts.

Supervision will be provided by a licensed clinical social worker (CP) who is deeply familiar with the community and was instrumental in the development of the intervention and a project manager on the team. External reviews of fidelity and intervention outcomes will be reviewed by the study PI and a licensed child psychiatrist who is on the study team.

### Pilot Study Procedures: Recruitment, Selection, and Consent Process

This study is approved by the institutional review board of Yale University and has been registered on Clinical Trials.gov (NCT07044752). The recruitment plan for the pilot study intervention will involve a collaborative partnership with a local high school, working closely with the principal and lead school counselors to identify youth who may benefit from participation. School counselors and teachers will play a key role in identifying students who exhibit signs of heightened anxiety or have been classified as high risk based on behavioral observations, academic performance, and self-reported concerns. To ensure a targeted and effective approach, recruitment will prioritize students who have been flagged by school guidance counselors for increased anxiety symptoms, difficulty managing stress, or other related mental health challenges. Once identified, eligible students will be invited to participate, with parental consent waived as per institutional review board approval. The intervention cohorts will be divided by sex (self-identified by the youth) to foster a comfortable and supportive group dynamic, allowing for tailored discussions and activities that align with the unique experiences and stressors faced by male and female students. This structured recruitment approach ensures that the intervention reaches those in greatest need while maintaining a supportive and inclusive environment for all participants. After referral to the intervention, the relevant information of the study will be provided to all participants in detail, and they will be given sufficient time to consider whether to participate in the study. If the participants agree to participate in the intervention, they will sign an informed assent form. Parental consent will be waived; however, all parents of youth participants will receive an information sheet indicating that their child is participating in an intervention. No blinding or randomization will occur in this phase of pilot testing. Due to participants being classed as a vulnerable population, they will also be notified of mandated reporter requirements. Each intervention group will consist of 5 to 10 participants, with a preferred group size of 8 with a total sample of 50 participants.

### Intervention Facilitators

The intervention will be facilitated by a license-eligible social worker who is on the study’s YAB and is from the community and a license-eligible clinical psychologist on the study team. Having facilitators from the community is crucial, as research indicates that community-based mentors and professionals foster stronger connections with participants, enhance engagement, and improve intervention outcomes [25,26]. These facilitators bring an intrinsic understanding of local stressors, social dynamics, and cultural nuances that external professionals may not fully grasp. Their presence helps to build trust and relatability, making the youth feel more comfortable discussing their experiences and applying coping strategies. Additionally, incorporating local facilitators into the program fosters sustainability, as they can continue to serve as mental health advocates within their community beyond the duration of the intervention [25,26]. This approach ensures that the program is not only evidence based but also deeply rooted in the strengths and needs of the community it serves.

### Study Measures: Baseline and Posttreatment Tests

The study will be conducted over 5 weeks with multiple assessment points depending on the measure. Outcome measures will be collected at the first session, which will be considered at baseline (T1) and after treatment (T2; 4 weeks from the baseline assessment or after the last intervention session has been completed).

### Primary Outcomes

The primary outcome is the mean change in anxiety scores on the Generalized Anxiety Disorder-7 (GAD-7) questionnaire for adolescents between the baseline, after the intervention, and at the 3-month follow-up (T2). The questionnaire has 7 items, each with a 4-point scale, with scores between 0 and 21 points. Scores are considered as follows: 0 to 4 symptoms (minimal), 5 to 9 symptoms (mild), 10 to 14 symptoms (moderate), and 15 to 21 symptoms (severe). The GAD-7 has demonstrated excellent psychometric properties [27-30]. The items inquire about the degree to which the patient has been bothered by feeling nervous, anxious, or on edge; not being able to stop or control worrying; worrying too much about different things; having trouble relaxing; being so restless that it is hard to sit still; becoming easily annoyed or irritable; and feeling afraid as if something might happen.

### Secondary Outcomes

Secondary outcomes will include the Coping Self-Efficacy Scale, a validated instrument assessing individuals’ confidence in using adaptive coping strategies to manage stressors [31]. Depression symptoms will be assessed using a validated measure such as the Patient Health Questionnaire-9 [32], which will allow us to explore whether reductions in depressive symptoms contribute to improvements in other targeted outcomes. By integrating these measures, we aim to comprehensively evaluate the intervention’s impact and identify key mechanisms driving behavior change.

### Sample Size and Power Justification

This study was powered based on the primary aim of assessing the feasibility of the newly developed intervention. Sample sizes ranging from 24 to 50 have been recommended for feasibility studies [31,32]. With a sample size of 50, we expect to retain at least 80% of the participants at 3 months after the intervention. For primary and secondary aims, we will estimate effect sizes from means and SDs, which will be used to determine the power needed for a future randomized controlled trial.

The planned analyses will include both descriptive and inferential statistics to assess the intervention’s effectiveness. The main outcome of interest will be anxiety. We expect that mean GAD-7 scores in the posttest assessments will be significantly lower than baseline scores. Baseline demographic characteristics, including age, sex, race, and socioeconomic status, will be reported. A repeated measures ANOVA within a within-subjects design will be used to test this hypothesis, with Shapiro-Wilk tests assessing normality assumptions. Based on previous literature that has used these measures, the distribution of difference scores will likely be normal. The study team will also explore whether changes in GAD-7 scores differ by sex. In addition, the study team will conduct statistical analyses to assess the reliability and construct validity of the GAD-7 questionnaire within the sample. Specifically, confirmatory factor analysis and Cronbach α will be used to evaluate the scale’s factor structure and internal consistency. Given previous use of the GAD-7 questionnaire in similar samples of youth of color [27,28], we anticipate that the scale will demonstrate strong psychometric properties in this population [29,30].

Additionally, logistic regression models will estimate the likelihood of clinically significant improvements in mental health and behavioral outcomes based on key predictors, including baseline symptom severity, intervention dosage, and demographic factors. Given the longitudinal nature of our data, we will use linear mixed-effects models to account for within-subject correlations and evaluate changes over multiple follow-up points. These models will help determine whether intervention effects are sustained and whether specific subgroups (eg, sex-based cohorts) exhibit differential responses.

### Fidelity to Intervention

A fidelity form created by the study team will be used to monitor fidelity to the intervention. Each facilitator will complete the fidelity form after each session. The form will track the main activities engaged in, skills covered, deviations from protocol, and adverse events that emerge during each session. Open-ended responses will be reviewed by the study team to identify any themes.

### Qualitative Analysis

Focus groups will be conducted after the final session with youth participants and after the baseline and posttreatment surveys have been completed. A study team member who was not involved in delivering the intervention will facilitate the groups to reduce bias and enhance transparency. Findings will offer deeper insight into the intervention’s effectiveness and acceptability, as well as how participants describe anxiety and its environmental influences in their lives.

### Ethical Considerations

To safeguard the confidentiality of the participants and to ensure proper handling of all data, processing of all data reporting complies with Collaborative Institutional Training Initiative recommendations and is in accordance with the established guidelines on ethical conduct in human research. Participants will provide written informed assent. All participant data will be allocated a deidentified code so that confidentiality is maintained during the study and when the data are stored. Deidentified data will be stored in password-protected secure online data storage repositories at Yale University. Only PIs, interventionists, the statistician, the project manager, and research assistants will have access to these deidentified data for the following purposes: (1) assessment of participants’ acute mental health, (2) quality improvement of the intervention, and (3) publication of findings. Any adverse events or clinical complications that arise as a result of the intervention will be reported by the interventions to the supervisor and PI after the session and during regular project supervision. All adverse events will be documented.

Due to participants being classed as a vulnerable population, they will also be notified of mandated reporter requirements during the assent process and at the beginning of each session. Considering that youth may share vulnerable personal information, students will be reminded of the importance of confidentiality at the beginning of each session. As participation will take place on school grounds, measures will also be taken to not reveal students’ participation in the group to nonparticipating peers. While students will be provided with resources at the end of each session, should a participant express high, acute mental health needs outside of the scope of the intervention, they will be referred to the school’s psychologist or social worker.

## Results

This pilot intervention is a part of a larger community-based study funded by the NIH. The study began in September 2020 and ended in August 2025 [21]. Recruitment for the pilot intervention trial began in April 2025, with the first session beginning in May 2025. In collaboration with a local high school in the city of Paterson. School counselors referred youths who were considered to display moderate to high levels of anxiety. The intervention was completed at the end of June 2025. The intervention took place during school hours, in a local classroom that was provided by the high school.

## Discussion

### Anticipated Findings

The study teams hypothesize that there will be a reduction of anxiety symptoms in youth who participate in the intervention. In addition, there may be notable differences by sex and how messages around anxiety are received. The significant psychosocial burden of anxiety and persistent resource accessibility gaps necessitates continued efforts to blend best practices in CBT with the unique needs faced by underresourced urban youth. This study will be testing out hypotheses around the application of CBT skills in addressing the underlying mechanisms that create and maintain anxiety for urban youth; that is, participants will be encouraged to actively identify and challenge unhelpful or unrealistic cognitions related to salient experiences (examinations and class presentations) and difficult interpersonal interactions. This process is complemented by behavioral activation to encourage broader engagement in coping skills and support seeking. Participants will also be encouraged to practice graded exposure to reduce the avoidance of participant-identified stressful situations, as well as problem-solving barriers to effectively engage in helpful coping (eg, seeking support when current coping skills are no longer effective). One major strength of the pilot intervention design is that the conceptualization of this intervention has had significant input from the population it aims to support: urban youth. It also tackles some of the frequent situations and target maladaptive cognitions the youth identified. While individual and group CBT-based anxiety interventions have been considered very useful in preventing and treating childhood anxiety [33], there is less research on using such interventions within a community-level participatory framework. We acknowledge the potential bias introduced by conducting theater testing with an existing YAB. However, we believe that the invaluable feedback provided by their participation mitigates this confounder. Findings from this community-based pilot study will be disseminated widely, including the local school where the intervention was implemented. Results will also be shared with community stakeholders through infographics, an end-of-year report to city officials, and on social media platforms (eg, Instagram) to ensure accessibility and transparency.

While a lack of randomization of this pilot study is a limitation, the decision to offer the intervention to eligible youth reflects our commitment to addressing an urgent community-identified need—particularly in a city where no formal anxiety-focused interventions currently exist. Future directions include scaling the intervention through a larger waitlist-controlled or randomized controlled trial and adapting it for implementation in other cities facing similar mental health disparities.

### Conclusions

This pilot study will provide a basis for the efficacy of a CBPR-guided, CBT-informed intervention for anxiety in school-aged urban adolescents. Outcomes from this study will provide evidence for the feasibility of incorporating this framework within urban communities and highlight the importance of researchers collaborating and cocreating solutions with youth and community members.

## Abbreviations

CBPR: community-based participatory research
CBT: cognitive behavioral therapy
GAD-7: Generalized Anxiety Disorder-7
NIH: National Institutes of Health
PI: principal investigator
YAB: youth advisory board
